# Fatal Human Case of Zika and Chikungunya Virus Co-Infection with Prolonged Viremia and Viruria

**DOI:** 10.3390/diseases6030053

**Published:** 2018-06-21

**Authors:** Kelly R. Silva, Blanca E. R. G. Bica, Eduardo S. Pimenta, Rodrigo B. Serafim, Mirhelen M. Abreu, Jorge L. S. Gonçalves, Larissa de S. Santana, Mauro J. Cabral-Castro, José M. Peralta, Marta G. Cavalcanti

**Affiliations:** 1 Serviço de Reumatologia, Hospital Universitário Clementino Fraga Filho, Universidade Federal do Rio de Janeiro (HUCFF/UFRJ), Rio de Janeiro 21941590, Brazil; kellyrgues@gmail.com (K.R.S.); blancaelena@hucff.ufrj.br (B.E.R.G.B.); mirhelen.abreu@gmail.com (M.M.A.); 2 Serviço de Doenças Infecciosas e Parasitárias, Hospital Universitário Clementino Fraga Filho, Universidade Federal do Rio de Janeiro (HUCFF/UFRJ), Rio de Janeiro 21941590, Brazil; dudupimenta85@gmail.com (E.S.P.); cavalcanti.marta66@gmail.com (M.G.C.); 3 Programa de Pós Graduação em Doenças Infecciosas e Parasitárias, Faculdade de Medicina, Universidade Federal do Rio de Janeiro (FM/UFRJ), Rio de Janeiro 21941590, Brazil; rodrigobserafim@gmail.com (R.B.S.); maurojorgerj@gmail.com (M.J.C.-C.); 4 Serviço de Clínica Médica, Hospital Universitário Clementino Fraga Filho, Universidade Federal do Rio de Janeiro (HUCFF/UFRJ), Rio de Janeiro 21941590, Brazil; 5 Instituto de Microbiologia Paulo de Góes, Universidade Federal do Rio de Janeiro (IMPG/UFRJ), Rio de Janeiro 21941902, Brazil; jorgelsg@gmail.com (J.L.S.G.); xlarissasantana@gmail.com (L.d.S.S.)

**Keywords:** Zika virus, Chikungunya virus, co-infection, systemic lupus erythematosus

## Abstract

Zika virus (ZIKV) infection usually presents as a mild and self-limited illness, but it may be associated with severe outcomes. We describe a case of a 30-year-old man with systemic erythematous lupus and common variable immunodeficiency who became infected with both Zika (ZIKV) and Chikungunya (CHIKV) virus during the 2016 outbreak in Rio de Janeiro, Brazil. The patient presented with intense wrist and right ankle arthritis, and ZIKV RNA and virus particles were detected in synovial tissue, blood and urine, and CHIKV RNA in serum sample, at the time of the diagnosis. During the follow up, ZIKV RNA persisted for 275 days post symptoms onset. The patient evolved with severe arthralgia/arthritis and progressive deterioration of renal function. Fatal outcome occurred after 310 days post ZIKV and CHIKV co-infection onset. The results show the development of severe disease and fatal outcome of ZIKV infection in an immunosuppressed adult. The data suggests a correlation between immunodeficiency and prolonged ZIKV RNA shedding in both blood and urine with progressive disease. The results also indicate a possible role for arbovirus co-infections as risk factors for severe and fatal outcomes from ZIKV infection.

## 1. Introduction

Arboviruses are among the more important emergent and re-emerging viruses in the world. The infections caused by Dengue (DENV), Zika (ZIKV) and Chikungunya (CHIKV) virus have similar clinical manifestations. Mostly, ZIKV infection lasts 2–7 days and manifests as an asymptomatic to a mild disease. However, ZIKV mono-infection can evolve into a severe and fatal disease in children and adults with increased rates of hospitalizations [1,2]. Neuropathies, uveitis, thrombocytopenia and, arthritis were previously reported as ZIKV atypical and severe clinical presentations [3,4]. Nonetheless, risk factors associated with poor disease outcome are still unclear [1].

In areas with arbovirus co-circulation, there is a great concern in the determination of the precise etiological diagnosis since these conditions have similar clinical manifestations [5]. However, immunological methods for detecting antibodies to ZIKV may show cross-reactivity with other flaviviruses, including DENV, which may hamper accurate diagnosis [6,7,8]. Therefore, molecular diagnosis, by detecting the presence of viral genetic material in a clinical sample, is considered the strongest evidence of causality [9,10,11].

Co-infections between two or three of these viruses have been reported in areas with arbovirus co-circulation [5,12,13,14,15]. Although ZIKV co-infections may evolve like mono-infections, they might also be implicated in progression to severe forms of the disease [2,16]. In addition, the role of co-infections in virus kinetics and its association to pathogenesis is not understood.

## 2. Case Presentation

**Figure 1:**
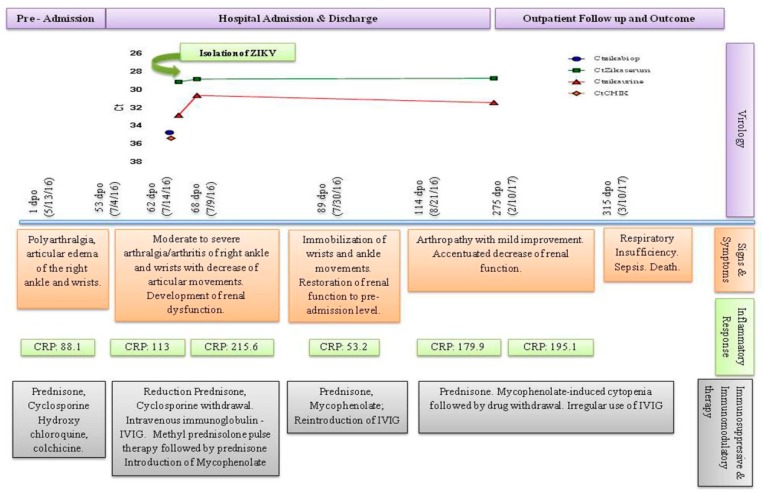
Timeline of virological kinetic, clinical features, inflammatory response and immunosuppressive/immunomodulatory therapy. Persistence of ZIKV RNA occurred in parallel with progressive clinical deterioration and increased inflammatory response during drug-induced immunosuppression. ZIKV replication was demonstrated in the first serum sample. Cycle threshold: Ct; reactive, if Ct < 38 and not reactive, >38. * Not reactive; DPO: Days post symptom onset; Reference values: CPR–0.0–5.0 mg/dL.

On 13 May 2016, a 30-year-old male presented with fever (39–40 °C) and headache followed by severe arthralgia and arthritis of both wrists, metacarpal, and phalangeal joints bilaterally, and the right ankle. Around five weeks later, the patient remained with persistent fever. And, amoxicillin-clavulanate was started to treat a lower respiratory tract infection with remission of symptoms although arthralgia/arthritis remained. Early July, the patient evolved again with recurrent fever and severe arthralgia/arthritis associated with loss of joint function without response to non-steroidal anti-inflammatory drugs (NSAIDs), being admitted to the hospital five days post new symptom onset (Figure 1). Informed written consent was obtained from the participant after approval by the Ethics in Research Committee of Hospital Universitário Clementino Fraga Filho, Federal University of Rio de Janeiro (reference CAAE 02920212.8.3001.5279).

The patient was diagnosed with systemic lupus erythematosus (SLE) at the age of 9; he was diagnosed with Nephritis class III/IV in 2007 and with a common variable immunodeficiency, in 2014. Despite previous irregular treatment with immunosuppressive and immunomodulatory therapy such as Rituximab (2008–2010), the patient was using Prednisone, Hydroxychloroquine and Cyclosporine in addition to Colchicine and Prophylactic Azithromycin at hospital admission (Figure 1).

Physical examination showed a temperature of 36.5 °C, redness, warmth, and swelling of left and right wrists and right ankle. Although there were no clinical manifestations of nephropathy, laboratory results showed a slight renal impairment. In addition, he presented anemia, leukocytosis with neutrophilia, lymphopenia and elevated inflammatory markers, such as C-reactive protein (CRP) (Table 1).

**Figure 2:**
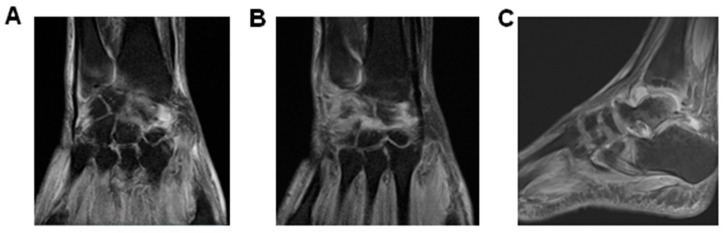
Development of severe tenosynovitis during arboviral coinfection. MRI of wrists and right ankle was performed 278 days post symptom onset to assess joint inflammatory evolvement showed in (**A**) (right wrist) and (**B**) (left wrist), synovitis and tenosynovitis bilaterally. Coronal images demonstrated the presence of edema and excessive fluid in carpus and radioulnar joints, and also in flexor and extensor compartments. (**C**) sagittal MRI Diffusion-Prepared (DP) weighted sequence with fat suppression image of the right ankle showing synovial overgrowth within the tibiotalar and intertarsal joints. Signs of plantar fasciitis and hyperintense abnormality were also observed, corresponding to a pattern of edema.

Because septic arthritis by gonococcal infection was initially suspected, ceftriaxone and azithromycin were used in addition to prednisone dose reduction and cyclosporine withdrawal (Figure 1). All hemocultures were negative and synovial liquid (SL) was not accessible by needle aspiration. Ultrasonography showed severe inflammatory joint disease (Table 2 and Figure 2). Because the right ankle also presented high inflammatory response, a synovial biopsy was indicated. Tissue fragment showed fibro-adipose overgrowth, hypervascularization and granulation tissue with mono and polymorphonuclear infiltrate and fibrin deposition, still suggesting infectious arthritis. No bacterial, fungal or *Mycobacterium tuberculosis* infections were detectable by direct examination or culture. However, viral etiology could not be excluded yet.

Arbovirus co-transmission increased markedly in Rio de Janeiro in 2016, and the patient’s mother and sister had a history of non-laboratory proved Chikungunya virus (CHIKV) and Zika virus (ZIKV) infection, respectively. Arthritogenic arboviruses was then investigated. Molecular diagnosis and ZIKV and CHIKV isolation in cells culture were performed in serum, urine and/or tissue samples. Viral RNA of serum, urine, and tissue were extracted from 250 μL of clinical samples using TRIzol LS Reagent (Ambion Inc, Foster City, CA, USA) according to the manufacturer’s recommendations. cDNA synthesis and real-time RT-PCR for detection of CHIKV and ZIKV RNA were performed by GoTaq 1-Step Probe RT-qPCR System (Promega Corporation, Madison, WI, USA) according to the manufacturer’s recommendations. The real-time RT-PCR protocol included specific primers and probe sets previously describe [9,10]. Biological samples showing amplification by real-time RT-PCR underwent culturing for viral isolation in cells of different lineage, such as *Macaca mulatta* kidney (LLC-MK2) and African green monkey kidney epithelial cells (VERO and MA-104). Commercial kits Anti-CHIKV IgM and IgG ELISA (Euroimmun Corporation, Luebeck, Germany) were used for detection of anti-CHIKV IgM, IgG, and the Dengue IgM Capture DxSelect, Dengue IgG DxSelect and Dengue NS1 antigen DxSelect (Focus Diagnostics, Diasorin Molecular LLCCypress, CA, USA) were used for the detection of IgM and IgG anti-DENV and NS1 antigens. CHIKV RNA amplification in the patient serum was detected on day 62 and low level of IgG anti-DENV was also demonstrated (Table 1 and Figure 1).

The renal function declined progressively, methylprednisolone pulse therapy and intravenous immunoglobulin (IVIG) were introduced along with mycophenolate. Renal function improved to pre-admission level in addition to a modest clinical and ultrasonographic improvement of the arthropathy (Table 1 and Table 2).

After hospital discharge, the patient had another admission and needed drug adjustment after drug-induced untoward effects. In addition, recurrent arthralgia and joint immobilization correlated with active inflammatory response confirmed by bone scintigraphy, USG and MRI (Table 2 and Figure 2).

Both SLE and/or viral-induced disease were investigated. After 275 days post symptoms onset, the patient still presented ZIKV RNA in both blood and urinary compartments (Figure 1). The patient died 28 days later of renal failure and pulmonary sepsis.

## 3. Discussion

Cases of arbovirus co-infections like ZIKV/DENV, ZIKV/CHIK, DENV/CHIKV, and even triple infections, have become notorious in different arbovirus co-transmission areas [5,12,13,14]. In the Americas, ZIKV fatal infections have been described, but data on their association with immunodeficiency is still limited [17]. Additionally, the role of co-infection in pathogenesis and clinical outcomes is poorly understood. The present work is the first report on ZIKV infection fatal outcome in the presence of CHIKV co-infection, SLE/CVID, and drug-induced immunosuppression. Detection of ZIKV RNA in synovial tissue is also uncommon and might represent a direct effect of ZIKV in the exacerbation of articular manifestations [4]. In addition, prolonged ZIKV viremia and viruria during drug-induced immunodeficiency coincided with the deterioration of clinical manifestations. Previous reports suggest that immune suppression and/or autoimmune disorders could be associated with severe illness [1].

In the present case, drug-induced decreased host immune response and/or uncontrolled SEL might be involved in disease severity, in combination with an exacerbated viral-induced disease. In this case, CHIKV RNA was also detected in a single serum sample, which could be related to the first onset of arthritic symptoms weeks before hospital admission. CHIKV RNA might persist for longer periods. In our experience, prolonged clinical manifestations are more often associated with ZIKV/CHIKV co-infection that with mono-infection. Although the persistence of ZIKV RNA shedding in different compartments has been described before, no clear correlation with disease progression was established, nor a risk factor determined [18]. In the present study, both co-infection and immunosuppression seem to overlap as risk factors for prolonged and severe clinical disease and persistent RNA in blood and urinary compartments.

ZIKA and CHIKV infections might be associated with the onset or exacerbation of arthralgia/arthritis in individuals with a history of co-morbidities and rheumatological disorders or co-infection and progression to fatal outcomes [2]. RNA sequencing was unavailable, and so ZIKV strain-specific-induced pathogenesis could not be ruled out. In this case, the extent of the role of virus-induced and SLE/CVID-associated pathogenesis in articular and renal disorders is still unclear. Renal function deterioration could result from the direct effect of ZIKV and/or CHIKV infection since renal tissues present virus particles. SLE nephritis could also be the major determinant of the fatal disease once it is related to increased fatality rates. Overall, SLE might cause arboviral co-infection-related morbidity/mortality and vice versa, both decisive in the fatal outcome of a ZIKV infection.

## Figures and Tables

**Table 1:** Laboratory findings and image features of Zika and Chikungunya co-infection in immunosuppressed systemic lupus erythematosus patient.

Laboratory Findings	Pre Hospital Admission	Hospital Admission		Follow Up			
	1–52 dpo	≥Day 53 dpo	>80 dpo	≥89 dpo	114–140 dpo	141–266 dpo	267–315 dpo
ZIKV RNA tissue		Ct 34.7					
ZIKV RNA serum		Ct 29.1	Ct 30.6				Ct 30.4
ZIKV RNA urine		Ct 32.8	Ct 28.8				Ct 28.7
CHIKV RNA serum		Ct 35.3					
ZIKV isolation		Positive					
CHIKV isolation		Negative					
DENV NS1		0.541	0.456				
Anti-DENV IgM		0.011	0.012				
Anti-DENV IgG		0.337	2.116				
Anti-CHIKV IgM		0.0 *	0.08				
Anti-CHIKV IgG		0.0 *	0.08				
BUN	100	88	118	89	69	73	119
Creatinine	3.40	2.50	3.50	2.20	2.40	2.80	2.60
Urinary Protein		254	137	115			
Proteinuria		3+	3+	3+	3+	3+	3+
RBC (× 106/mm^3^)	4.21	3.30	3.26	4.06	3.65	2.78	2.41
Hematocrit (%)	36	8.80	8.90	11.20	9.50	7.70	6.10
Hemoglobin (g/dL)	11.8	26.40	27.60	34.00	30.00	33.60	18.90
WBC (mm^3^)	10,800	12,600	9300	9000	6800	2300	3900
Neutrophils (mm^3^, %)	7884 (73)	9941 (79)	7254 (78)	8109 (90)	4964 (73)	1127 (49)	2652 (68)
Lymphocytes (mm^3^, %)	2052 (19)	1235 (9.8)	1395 (15)	594 (6.6)	1156 (17)	943 (41)	975 (25)
Platelets	165,000	380,000	249,000	252,000	240,000	211,000	8700
IgA				<25			
IgE				<18			
IgG				267			
IgM				<17			
ANA							Negative

Abbreviations and reference values: ANA: Anti-nuclear antibody; BUN: 10–50 mg/dL; Creatinine: 0.8–1.3 mg/dL; Cycle threshold: Ct; reactive if Ct < 38 and not reactive >38. * Not reactive; DPO: days post symptom onset; IgA: 70–400 mg/dL; IgE: <100 UI/mL; IgG: 700–1600 mg/dL; IgM: 40–230 mg/dL; Proteinuria: trace; 1+; 2+; 3+ & 4+; Urinary Protein: 0–119 mg/dL; RBC: Red Blood Cells; WBC: White Blood Cells.

**Table 2:** Imaging studies of arboviral arthritis in SLE.

	Hospital Admission	Follow Up
USG	≥Day 53dpo (7/4/16) Wrists: Tenosynovitis with severe SP. PDIII Right Ankle: Moderate SP; PD II	Day141-266 dpo (9/29/16–2/1/17) Wrists: Moderate to severe SP. PD II to III Right Ankle: Mild to Moderate SP; PD I
BSc		Day 114–140 dpo ((9/2–9/28/16) Prominent localized inflammation in the right and left wrists and right ankle.
MRI		Day 114–140 dpo (9/2–9/28/16) Right Ankle\Subchondral areas of talo-tibial, talo-calcaneus, navicular bone and Lisfranc & Chopard presented edema and inflammatory process compatible with synovitis. Liquid infiltration of foot plantar, abductors and flexor muscles. Extensive edema and inflammatory infiltration of soft tissues around the right ankle. Day 267–315dpo (2/2–3/10/17) Wrists: Test revealed synovitis and tenosynovitis. A moderate increase of intra-articular fluid. Augmented tendon thickening and intercarpal arthrosis. Marked periosteal inflammation and oedematous capitis with carpi synovitis.

Abbreviations: BSc: Bone Scintigraphy; DPO: Day post onset; MRI: Magnetic Resonance Image; PD: Power Doppler; SP: Synovial Proliferation; USG: Ultrasonography.

## References

[1] Azevedo R.S., Araujo M.T., Martins Filho A.J., Oliveira C.S., Nunes B.T., Cruz A.C., Nascimento A.G., Medeiros R.C., Caldas C.A., Araujo F.C. (2016). Zika vírus epidemic in Brazil. I. Fatal disease in adults: Clinical and laboratorial aspects. J. Clin. Virol..

[2] Zonneveld R., Roosblad J., Staveren J.W., Wilschut J.C., Vreden S.G., Codrington J. (2016). Three atypical lethal cases associated with acute Zika virus infection in Suriname. IDCases.

[3] Da Silva I.R.F., Frontera J.A., Bispo de Filippis A.M., Nascimento O.J.M.D., RIO-GBS-ZIKV Research Group (2017). Neurologic complications associated with the Zika Virus in Brazilian adults. JAMA Neurol..

[4] Roimicher L., Ferreira O.C., Arruda M.B., Tanuri A. (2017). Zika Virus in the joint of a patient with rheumatoid arthritis. J. Rheumatol..

[5] Villamil-Gómez W.E., Rodriguez-Moeales A.J., Uribe-Garcia A.M., González-Arismendy E., Castellanos J.E., Calvo E.P., Álvares-mon M., Musso D. (2016). Zika, Dengue and Chikungunya co-infection in a pregnant woman from Colombia. Int. J. Infect. Dis..

[6] Nunes M.R.T., Faria N.R., de Vasconcelos J.M., Golding N., Kraemer M.U., de Oliveira L.F., da Silva Azevedo R.d.S., Andrade da Silva D.E., Pinto da Silva E.V., da Silva S.P. (2015). Emergence and potential for spread of Chikungunya virus in Brazil. BMC Med..

[7] Campos G.S., Bandeira A.C., Sardi S.I. (2015). Zika Virus Outbreak, Bahia, Brazil. Emerg. Infect. Dis..

[8] Kelser E.A. (2016). Meet dengue’s cousin, Zika. Microbes Infect..

[9] Lanciotti R.S., Kosoy O.L., Laven J.J., Panella A.J., Velez J.O., Lambert A.J., Campbell G.L. (2007). Chikungunya virus in US travelers returning from India, 2006. Emerg. Infect. Dis..

[10] Lanciotti R.S., Kosoy O.L., Laven J.J., Velez J.O., Lambert A.J., Johnson A.J., Stanfield S.M., Duffy M.R. (2008). Genetic and serologic properties of Zika virus associated with an epidemic, Yap State, Micronesia, 2007. Emerg. Infect. Dis..

[11] Waggoner J.J., Pinsky B.A. (2016). Zika virus: Diagnostics for an emerging pandemic threat. J. Clin. Microbiol..

[12] Dupont-Rouzeyrol M., O’Connor O., Calvez E., Daurès M., John M., Grangeon J.P., Gourinat A.C. (2015). Co-infection with Zika and Dengue Viruses in 2 Patients, New Caledonia, 2014. Emerg. Infect. Dis..

[13] Zambrano H., Waggoner J.J., Almeida C., Rivera L., Benjamin J.Q., Pinsky B.A. (2016). Case Report: Zika Virus and Chikungunya Virus CoInfections: A Series of Three Cases from a Single Center in Ecuador. Am. J. Trop. Med. Hyg..

[14] Kutsuna S., Kato Y., Nakayama E., Taniguchi S., Takasaki T., Yamamoto K., Takeshita N., Hayakawa K., Kanagawa S., Ohmagari N. (2017). A case of consecutive infection with Zika virus and Chikungunya virus in Bora Bora, French Polynesia. J. Infect. Chemother..

[15] Villamil-Gómez W.E., González-Camargo O., Rodriguez-Ayubi J., Zapata-Serpa D., Rodriguez-Morales A.J. (2016). Dengue, chikungunya and Zika co-infection in a patient from Colombia. J. Infect. Public Health.

[16] Sardi S.I., Somasekar S., Naccache S.N., Bandeira A.C., Tauro L.B., Campos G.S., Chiu C.Y. (2016). Coinfections of Zika and Chikungunya viruses in Bahia, Brazil, identified by metagenomic next-generation sequencing. J. Clin. Microbiol..

[17] Sarmiento-Ospina A., Vasquez-Serna H., Jimenez-Canizales C.E., Villamil-Gomez W.E., Rodriguez-Morales A.J. (2016). Zika virus associated deaths in Colombia. Lancet Infect. Dis..

[18] Paz-Bailey G., Rosenberg E.S., Doyle K., Munoz-Jordan J., Santiago G.A., Klein L., Perez-Padilla J., Medina F.A., Waterman S.H., Gubern C.G. (2017). Persistence of Zika Virus in Body Fluids-Preliminary Report. N. Engl. J. Med..

